# The Inhibition of Inflammasome by Brazilian Propolis (EPP-AF)

**DOI:** 10.1155/2013/418508

**Published:** 2013-04-16

**Authors:** Juliana I. Hori, Dario S. Zamboni, Daniel B. Carrão, Gustavo Henrique Goldman, Andresa A. Berretta

**Affiliations:** ^1^Departmento de Biologia Celular, Molecular e Bioagentes Patogênicos, Faculdade de Medicina de Ribeirão Preto, Universidade de São Paulo, FMRP/USP, 14049-900 Ribeirão Preto, SP, Brazil; ^2^Apis Flora Industrial e Comercial Ltda, Rua Triunfo, 945, 14020-670 Ribeirão Preto, SP, Brazil; ^3^Departamento de Ciências Farmacêuticas, Faculdade de Ciências Farmacêuticas de Ribeirão Preto, Universidade de São Paulo, Avenida do Café s/n., 14049-900 Ribeirão Preto, SP, Brazil; ^4^Laboratório Nacional de Ciência e Tecnologia do Bioetanol—CTBE, Caixa Postal 6170, 13083-970 Campinas, SP, Brazil

## Abstract

Propolis extracts have gained the attention of consumers and researchers due to their unique chemical compositions and functional properties such as its anti-inflammatory activity. Recently, it was described a complex that is also important in inflammatory processes, named inflammasome. The inflammasomes are a large molecular platform formed in the cell cytosol in response to stress signals, toxins, and microbial infections. Once activated, the inflammasome induces caspase-1, which in turn induces the processing of inflammatory cytokines such as IL-1**β** and IL-18. So, to understand inflammasomes regulation becomes crucial to treat several disorders including autoinflammatory diseases. Since green propolis extracts are able to regulate inflammatory pathways, this work purpose was to investigate if this extract could also act on inflammasomes regulation. First, the extract was characterized and it demonstrated the presence of important compounds, especially Artepillin C. This extract was effective in reducing the IL-1**β** secretion in mouse macrophages and this reduction was correlated with a decrease in activation of the protease caspase-1. Furthermore, we found that the extract at a concentration of 30 **μ**g/mL was not toxic to the cells even after a 18-hour treatment. Altogether, these data indicate that Brazilian green propolis (EPP-AF) extract has a role in regulating the inflammasomes.

## 1. Introduction

Over the last few decades, interest in natural medicines has been growing fast, leading to the discovery of new functional components and products that may help preventing or treating diseases. In this context, propolis extracts have gained special attention of consumers and researchers, due to their unique chemical compositions and functional properties [1, 2].

Propolis is a resinous material collected by bees (*Apis mellifera* L.) from exudates and buds of plants and mixed with wax and bee enzymes [1]. More than 300 compounds, among polyphenols, terpenoids, steroids, sugar, and amino acids, have been detected in raw propolis. Their abundance is influenced by geographical factors and botanical origins, as well as by collection season [3]. In this context, green propolis is only obtained from Brazil and its most important plant source is *Baccharis dracunculifolia* D.C. (Asteraceae) [4]. Several works reported biological activities to green propolis such as antiulcer [4, 5], anti-inflammatory [6], immunomodulatory activity (Machado et al., 2012, in press), antimutagenic [7], antifungal/antibacterial [3, 8], wound healing [9], and anti-*Candida albicans* [10].

Considering all these biological properties of propolis, the anti-inflammatory effect is one of the most well known. Reis et al. evaluated the anti-inflammatory activity of propolis standardized extract on edema induced by carrageenan, dextran, and histamine. The extract showed effective results at 650 mg/kg, significantly inhibiting the inflammatory process induced by carrageenan and histamine, but not by dextran [11]. The anti-inflammatory results were corroborated by Barros et al. (2007) that evaluated the effects in the models of gastric damage induced by ethanol, indomethacin, and stress in rats [4]. Paulino et al. demonstrated that green propolis extract, at low concentrations, induced anti-inflammatory and analgesic effects in mouse models, results obtained by oral or intraperitoneal administration [6]. Although propolis components responsible for the pharmacological activities are currently unknown, the flavonoids pinobanksin and kaempferol and the phenolic acid, artepillin C are strong candidates.

Some authors related that the biological activities of green propolis are mostly due to its high levels of prenylated *p*-coumaric acids derivatives, mainly 3,5-diprenyl-4-hydroxycinnamic acid (Artepillin C) [2]. In this context, Paulino et al. studied its anti-inflammatory effects, absorption, and bioavailability in mice model. *In vivo* results showed that Artepillin C reduced paw edema (38% in 6 hours) and decreased the number of neutrophils during peritonitis and prostaglandin E_2_. Moreover, *in vitro* results demonstrated decrease in nitric oxide production and NF-kB activity, suggesting an anti-inflammatory effect of propolis extract [6].

Recently, a complex of molecules that is important in inflammatory processes, named inflammasome, has been described [12–16]. The inflammasomes are a large multimeric complexes formed in the cell cytosol in response to stress signals, toxins, and microbial infections [15, 16]. After assembly of the multimeric complex, the inflammasome induces the activation of caspase-1 protease. Once activated, caspase-1 induces the processing of inflammatory cytokines, such as IL-1*β* and IL-18 [16, 17]. Moreover, caspase-1 activation also induces a type of inflammatory cell death named pyroptosis, thereby contributing to the generation of a proinflammatory response [18–20]. There are four inflammasomes described so far, distinguished by the receptor or NLRs (Nod-Like Receptors) involved in the complex: the NLRP1b [21, 22], the NLRC4/NAIP5 [23–25], the NLRP3 [26, 27], and AIM2 [28] inflammasomes.

The inflammasome activation is crucial for host defense to pathogens, but recent research has also found a role for the inflammasome in the pathogenesis of several diseases with an inflammatory component, such as type 2 diabetes, inflammatory bowel disease, and atherosclerosis [29–31]. These autoinflammatory diseases are clinical disorders that present recurrent inflammation due to abnormally increased inflammation mediated by cells of the innate immune system. However, the understanding of the inflammasomes regulation is still not clear and additional progress in this research field could contribute to new strategies in treating autoimmune diseases and their complication. Since propolis extract has been shown to regulate inflammatory pathways, it was investigated if the propolis standardized extract (EPP-AF) could also act on inflammasomes regulation.

## 2. Material and Methods

### 2.1. Collection and Preparation of Propolis Standardized Extract (EPP-AF)

The Propolis Standardized Extract (EPP-AF) was produced from a blend composed by propolis raw material obtained from several sites of Brazil according to previous standardization (Patent no. PI 0405483-0, published in Revista de Propriedade Industrial n. 1778 from 01/02/2005), the composition of the blend is majority constituted by green propolis. The extract was industrially produced and kindly provided by Apis Flora Company (Ribeirão Preto, SP, Brazil). Briefly, propolis was kept in −20°C for 12 h, ground to a fine powder in a blender. Then, it was extracted using hydroalcoholic solution (7 : 3), with dynamic maceration, during 72 h on room temperature, followed by percolation and a filtration process using, in the first step, the propolis biomass and secondly a 220 mesh stainless steel industrial line filter. Propolis extract obtained presents 11% w/v of dry matter and chemical composition standardized qualitatively and quantitatively by RP-HPLC.

### 2.2. Chemical Characterization of Propolis Extract

The propolis extracts were analyzed by HPLC using a Shimadzu apparatus equipped with a CBM-20A controller, an LC-20AT quaternary pump, an SPD-M 20A diode-array detector, and Shimadzu LC solution software. A Shimadzu Shim-Pack CLC-ODS column (4.6 mm × 250 mm, particle diameter of 5 *µ*m, pore diameter of 100 Å) was used. The mobile phase consisted of methanol (B), and a solution of water-formic acid (0.1% v/v), pH 2.7 (A). The method consisted of a linear gradient of 20–95% of B over a period of 77 minutes at a flow rate of 0.8 mL/min. Detection was set at 275 nm. For analysis it was used the standards caffeic, *p*-coumaric, and *trans*-cinnamic acids (Sigma-Aldrich, São Paulo, Brazil); artepillin C (Wako Pure Chemical Industries Co., Osaka, Japan), caffeic acid phenethyl ester (Sigma-Aldrich, São Paulo, Brazil), and aromadendrin-4′-O-methyl ether, isolated by de Sousa et al. and kindly donated by the authors [32]. Methanol HPLC-grade was obtained from J. T. Baker and water was treated in Milli-Q water purification system. All other chemicals were of reagent grade and were used without further purification. Propolis extract was diluted with 5 mL of methanol (HPLC grade) in 10 mL volumetric flasks, subjected to sonication for 10 min and filled to volume with Milli-Q water. The samples (*n* = 3) were filtered through a 45 *µ*m filter before analysis.

### 2.3. Bacterial Strain

The bacterium used in this study was Lp02 strain of *L. pneumophila* [33]. The bacteria were cultured in MOPS buffered charcoal-yeast extract (CYE) agar (1% yeast extract, 1% 3-(N-morpholino) propanesulfonic acid (MOPS), pH 6.9, 3.3 mM L-cysteine, 0.33 mM Fe(NO_3_)_3_, 1.5% Bacto agar, and 0.2% activated charcoal) and supplemented with thymidine (100 mg/mL) at 37°C. Before infection, bacteria were resuspended in sterile water and diluted to a multiplicity of infection (MOI) of at least 10, based on optical density (OD_600_).

### 2.4. Mice and Macrophage Preparation

C57BL/6 and Caspase-1^−/−^ mice were maintained and breed in Institutional Animal Facilities of the University of São Paulo. Bone Marrow Derived Macrophages (BMDMs) were prepared as previously described [34]. Briefly, bone marrow cells from femurs of adult mice were cultured for 7 days in RPMI 1640 containing 20% fetal bovine serum (FBS) and 30% L-929 cell conditioned media (LCCM). Macrophages were replated one day prior to infection and maintained at 37°C, 5% CO_2_, in RPMI 1640 media containing 10% FBS and 5% LCCM.

### 2.5. Cytotoxicity Assay

The propolis cytotoxicity was assayed by ethidium bromide (EtBr) staining as described previously [20]. In this assay, 2.0 × 10^5^ BMDMs were plated on 13 mm glass coverslips in 24-well tissue culture dishes for 16 h at 37°C, 5% CO_2_. The propolis was added on macrophages at 30, 100, or 300 *μ*g/mL during 1, 3, 6, or 18 hours. After the different times, the coverslips were inverted onto a 5-*μ*L drop of PBS containing 25 *μ*g/mL EtBr and 5 *μ*g/mL acridine orange. All cells were stained with acridine orange, whereas only cells with membrane pores allowed diffusion of EtBr into the cell. Pore-forming activity was measured as the percentage of BMDMs that stained positive with EtBr. Images were acquired using a Leica microscope (DMI4000B) with 10× and 40× objectives and analyzed using *ImageJ software* (TreeStar).

### 2.6. Cytokine Measurements

For cytokine determination, 2.0 × 10^5^ BMDMs were plated in 24-well plates for 16 h at 37°C, 5% CO_2_. For experiments with nigericin, gramicidin, and ATP, cells were pretreated with LPS (1 *μ*g/mL, Sigma-Aldrich, São Paulo, Brazil) for 4 h, treated with 30, 100, or 300 *μ*g/mL of propolis for 1 h and then added nigericin (20 *μ*m, Sigma-Aldrich, São Paulo, Brazil), gramicidin (100 *μ*g/mL, Sigma-Aldrich, São Paulo, Brazil), and ATP (5 mM, Sigma-Aldrich, São Paulo, Brazil) for additional 1 h. For bacterial experiments, macrophages were treated with 30, 100, or 300 *μ*g/mL of propolis for 1 h and infected with *L. pneumophila *at a MOI of 10 for additional 12 h. The cytokine in the supernatant was measured by enzyme-linked immunosorbent assay (ELISA) with a mouse IL-1*β* kit (BD OptEIA) according to the manufacturer's instructions.

### 2.7. Flow Cytometry Analysis of Endogenous Caspase-1

For analysis of active caspase-1 in BMDMs, 5.0 × 10^5^ macrophages were plated in 48-well plates, BMDMs were stimulated with LPS (1 *μ*g/mL, Sigma-Aldrich, São Paulo, Brazil) during 4 h, treated with 30 *μ*g/mL of propolis for 1 h and then added nigericin (20 *μ*m, Sigma-Aldrich, São Paulo, Brazil) for additional 1 h. Before being stained, macrophages were removed with cold phosphate buffered saline (PBS). Macrophages were stained for 1 h with FAM-YVAD-fluoromethylketone (FAM-YVAD-FMK; Immunochemistry Technologies) as recommended by the manufacturer. Data were acquired on a FACS-CantoII (Becton Dickinson) and were analyzed using *ImageJ software* (TreeStar).

### 2.8. Colony Forming Unit Assay (CFU)

To measure the number of bacteria in BMDMs, macrophage cultures were lysed in sterile water and cell lysates were combined with cell culture supernatant from the respective well. Lysates plus supernatants from each well were diluted in water, plated on CYE agar plates supplemented with thymidine, and incubated for 96 hours at 37°C for CFU determination.

### 2.9. Statistical Analysis

Statistical analyses were performed using GraphPad Prism, version 5.0, software. Data are expressed as the mean ± standard deviation (SD) and statistical significance calculated by two-way analysis of variance (ANOVA), followed by Bonferroni posttest analysis. Differences were considered statistically significant if the  *P*  value was <0.05.

## 3. Results

### 3.1. Chemical Characterization of Propolis Extract (EPP-AF)

**Figure 1:**
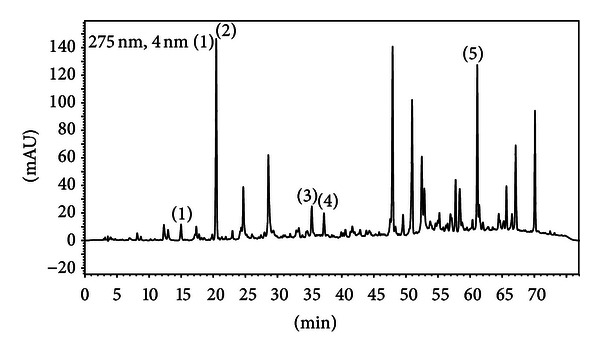
Fingerprint analysis of propolis alcoholic extract (EPP-AF) with four years and half of shelf-life stability conditions. Chromatograms were plotted at 275 nm, using RP-HPLC equipment with C18 (shim-pack, CLC-ODS (M), 25 cm × 4.6) column and gradient elution with methanol and acidic water (pH = 2.7). Chromatographic profile includes the compounds: (1) caffeic acid (around 15 min), (2) *p*-coumaric acid (around 20 min), (3) *trans*-cinnamic acid (around 35-36 min), (4) aromadendrin (38 min), and (5) Artepillin C (around 61-62 min).

**Figure 2:**
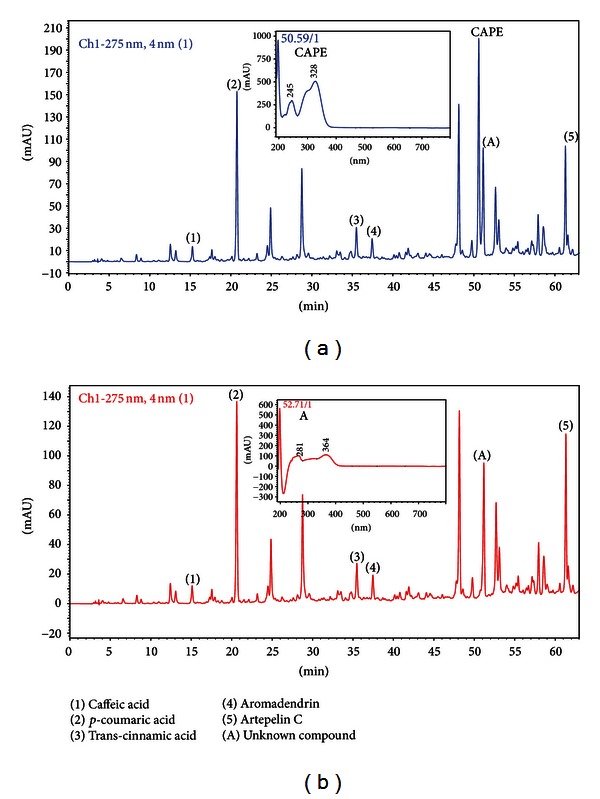
Fingerprint analysis of propolis standardized extract (EPP-AF) in comparison with enrichment sample with CAPE. (a) Presentation of propolis standardized extract (EPP-AF) with CAPE (100 *μ*g) in the same chromatographic conditions used in Figure 1, (b) propolis standardized extract showing a possible candidate to CAPE (A). To check the signs with similar retentions time of CAPE, UV spectra of CAPE and the candidate present in (b) were shown, demonstrating that propolis used does not present CAPE.

Propolis standardized extract (EPP-AF) was evaluated by HPLC and the fingerprint is represented on Figure 1. The results showed the presence of caffeic (1), *p*-coumaric (2) and *trans*-cinnamic (3) acids, the flavonoid aromadendrin (4) and the prenylated compound artepillin C (Figure 1) with respective values presented in Table 1 (mg/g). As a note, our results clearly showed that Brazilian propolis does not present CAPE (Caffeic Acid Phenethyl Ester), which has already been described having anti-inflammatory activities (Figure 2). Table 1 presents the quantitative characterization of propolis extract evaluated in the present work (Table 1).

### 3.2. The Effect of Propolis on IL-1*β* Secretion by Macrophages

**Figure 3:**
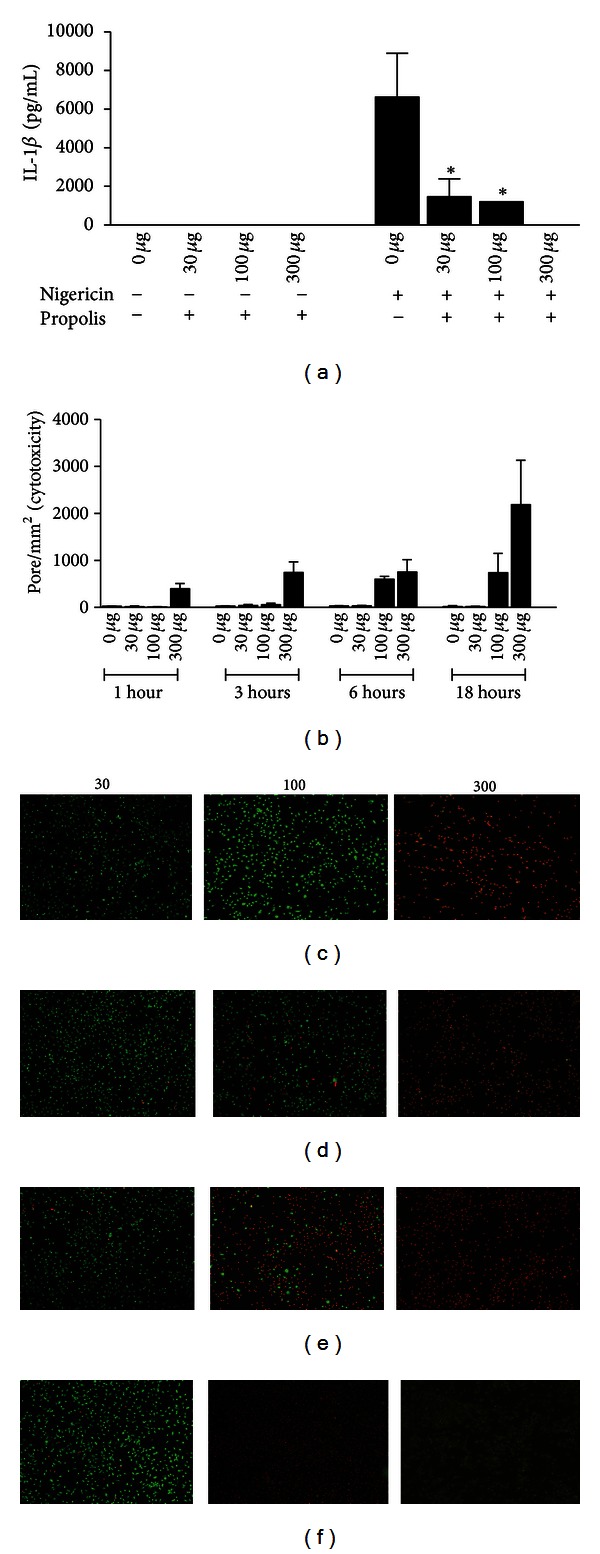
Propolis standardized extract (EPP-AF) reduces the secretion of IL-1*β* by mouse macrophages. (a) BMDMs from C57BL/6 mice were stimulated with LPS, pretreated or not with different concentrations of propolis extract and treated with nigericin for 1 h. The cell supernatant was collected and levels of IL-1*β* were measured by ELISA assay. (b) Cytotoxicity assay of propolis. BMDMs from C57BL/6 mice were treated with 30, 100, or 300 ug/mL of propolis during 1 h (c), 3 h (d), 6 h (e), and 18 h (f) and cells were analyzed by fluorescence microscopy. Data show average ± standard deviation and an asterisk indicates a  *P*  value of <0.05.

 Several studies have reported the anti-inflammatory and immunomodulatory properties of Brazilian propolis [3, 4, 35–37]. Recently, the strong anti-inflammatory effect of propolis has been reported regarding the inflammatory response in different experimental models, such as local and systemic models employing mice (Machado et al., 2012, in press). In the present work, it was investigated if propolis was also related with the modulation of the IL-1*β*, an essential cytokine involved in regulating inflammatory responses to both infectious and sterile injury [38–40]. To test this, we employed a canonical model with LPS and nigericin to induce IL-1*β* secretion by macrophages [41, 42]. Thus, BMDMs from C57BL/6 mice were prestimulated with LPS during 4 hours, to induce pro-IL-1*β*, and then treated with different concentrations of propolis (30, 100, or 300 *µ*g/mL) for 1 hour, followed by nigericin treatment for additional 1 hour. We observed that LPS treatment or LPS plus propolis did not induce the IL-1*β* secretion. However, there was an expressive secretion of IL-1*β* by macrophages that were treated with LPS and nigericin (Figure 3(a)). Of note, this cytokine was extremely reduced in macrophages that were treated with propolis before the addition of nigericin (Figure 3(a)).

We investigated if the observed reduction of IL-1*β* was not due to a cytotoxicity caused by propolis to the macrophages. Thus, BMDMs were treated with 30, 100, or 300 *µ*g/mL of propolis for 1, 3, 6, and 18 hours and BMDM viability was analysed by ethidium bromide (EtBr) incorporation, using fluorescence microscopy. The intact membranes fail to internalize EtBr, while cells containing pores or a rupture in plasma membranes become permeable to this dye [43]. This pore formation assay was performed by using EtBr in combination with acridine orange, a nonselective acidophilic green dye that stains both permeabilized and intact cells and therefore allows the determination of the percentage of cell death [44]. The results showed that after 1-hour treatment, the propolis concentrations of 30 and 100 *µ*g/mL were not toxic to the cells, while 300 *µ*g/mL was toxic to BMDMs (Figures 3(b) and 3(c)). By contrast, the use of propolis at 30 *µ*g/mL was not toxic even after 18 hours of treatment (Figures 3(b), 3(c), 3(d), 3(e), and 3(f)), which led to the choice of this dose for further experiments. The data presented, therefore, indicate that propolis can modulate the secretion of the inflammatory cytokine IL-1*β* by BMDMs, a feature solely dependent on the inflammasome functions.

### 3.3. Propolis Inhibits the NLRP3 Inflammasome

The cytokine IL-1*β* is expressed as a proprotein (pro-IL-1*β*) by immune cells and its activation requires a proteolytic processing in its mature form (IL-1*β*) by the protease caspase-1. However, caspase-1 is active only in the presence of activation signals, which in turn, takes place in the recently identified multimolecular complexes, called inflammasomes [14, 21]. In this regard, it was investigated if the modulation of IL-1*β* secretion by propolis was related with the modulation of the inflammasome. Since nigericin is a classical activator of caspase-1 via the NLRP3 inflammasome, we used nigericin to activate the canonical NLRP3 inflammasome. We monitored the endogenous caspase-1 activation in BMDMs treated with LPS, nigericin and propolis by staining BMDMs with a fluorescent dye that binds with high affinity to the active form of caspase-1 [45, 46].

**Figure 4:**
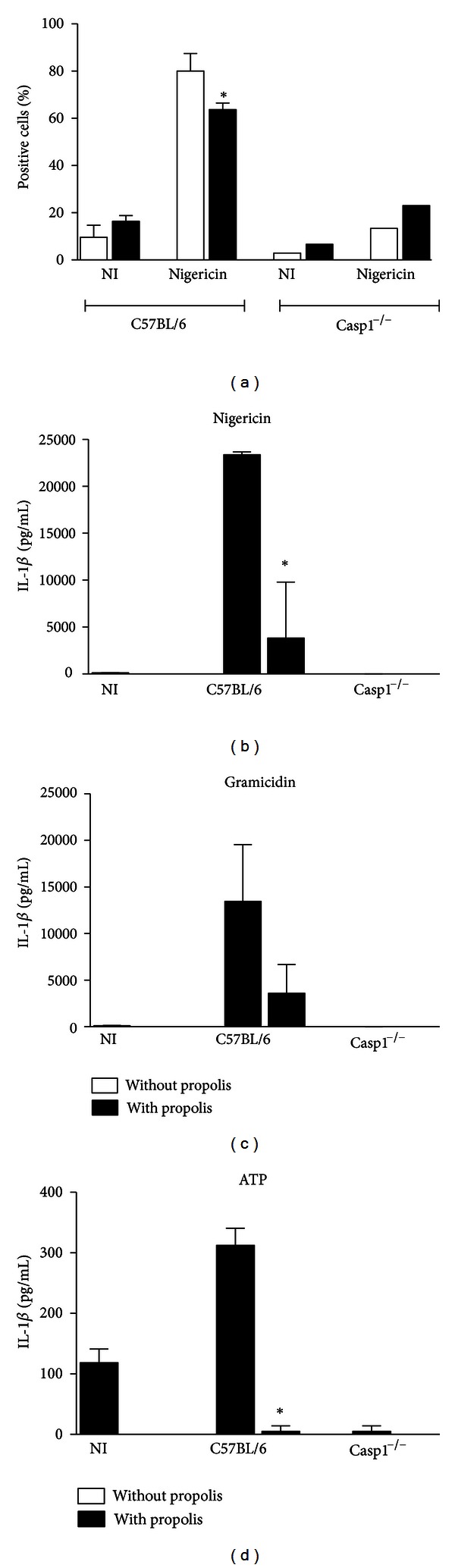
Propolis standardized extract (EPP-AF) inhibits the NLRP3 inflammasome. (a) BMDMs, 5.0 × 10^5^ macrophages were plated in 48-well plates, stimulated with LPS, pretreated or not with 30 ug/mL of propolis extract, and treated with nigericin for 1 h. The cells were stained for 1 h with FAM–YVAD–fluoromethylketone (FAM–YVAD–FMK) and analyzed by Flow Cytometer on a FACS-CantoII. 30.000 events were acquired. (b) BMDMs from C57BL/6 and Caspase-1^−/−^ mice were stimulated with LPS, pretreated or not with 30 ug/mL of propolis extract, and treated with nigericin, Gramicidin (c), and ATP (d) for 1 h. The supernatant was collected and levels of IL-1*β* were measured by ELISA assay. Data show average ± standard deviation and an asterisk indicates a  *P*  value of <0.05.

After the treatment with LPS and nigericin, a larger proportion of C57BL/6 macrophages stained positive for active caspase-1, while a significant reduction was observed in macrophages that were pretreated with propolis (Figure 4(a)). Very few BMDMs deficient for caspase-1 (Casp1^−/−^) presented FAM-YVAD staining after LPS and nigericin treatment, confirming that FAM-YVAD staining required caspase-1 (Figure 4(a)). These results indicate that propolis interferes with inflammasome platform impairing the caspase-1 activation by these complexes.

Finally, it was investigated the propolis action using different activators of the NLRP3 inflammasomes, such as ATP and gramicidin [47, 48]. It was observed that propolis inhibits the IL-1*β* secretion in BMDMs treated with the different activators of the NLRP3 (Figures 4(b), 4(c), and 4(d)), thus supporting the findings that propolis can inhibit the NLRP3 inflammasome.

### 3.4. Propolis Reduces the IL-1*β* Secretion in Mouse Macrophages Infected with *Legionella pneumophila*

Genetic studies in mice can distinguish so far four different inflammasomes: NLRP1b, NLRP3, NLRC4, and AIM2. Since it was observed the effect of propolis on NLRP3 inflammasome, it was also examined its action in another well-described inflammasome: the NLRC4 inflammasome.

**Figure 5:**
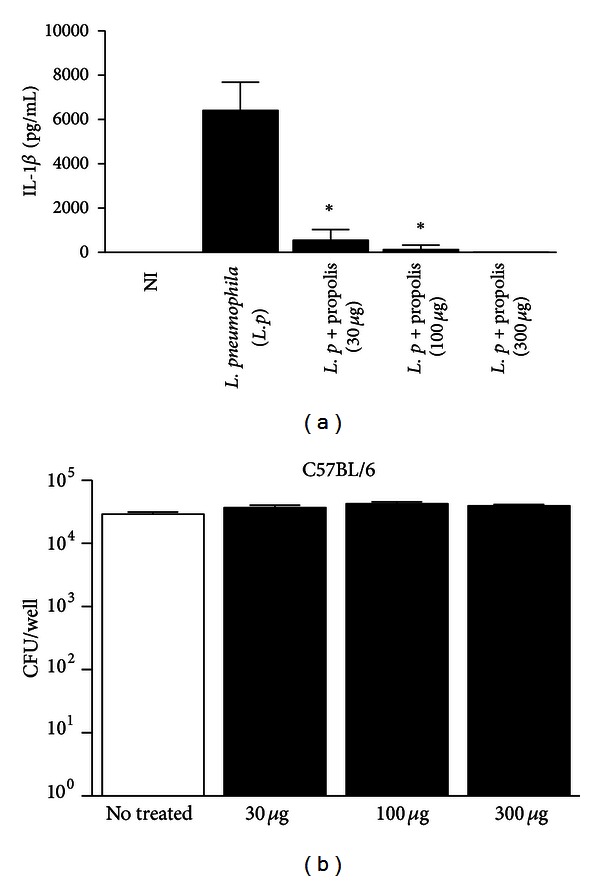
Propolis standardized extract (EPP-AF) reduces the IL-1*β* secretion in macrophages infected with *L. pneumophila*. (a) BMDMs from C57BL/6 mice were stimulated with LPS, pretreated or not with 30, 100, or 300 *μ*g/mL of propolis extract, and infected with *L. pneumophila *during 12 hours. The supernatant was collected and levels of IL-1*β* were measured by ELISA assay. (b) BMDMs from C57BL/6 mice were pretreated or not with 30, 100, or 300 *μ*g/mL of propolis extract and infected with *L. pneumophila*. Cultures were infected with 2 × 10^6^ bacteria and further incubated for 12 hours. After this period, the cells were lysate with deionized water and the bacteria were plated on CYE agar for CFU determination. Data show average ± standard deviation and an asterisk indicates a  *P*  value of <0.05.

The NLRC4 inflammasome responds to gram-negative bacterial components such as flagellin and basal body Rod proteins present in bacterial type III secretion systems [23, 24, 49]. Consequently, intracellular pathogens expressing these factors, such as *Salmonella typhimurium, Shigella flexneri, Pseudomonas aeruginosa, Burkholderia thailandensis*, and *Legionella pneumophila* can activate the NLRC4 inflammasome [15, 23, 24, 26, 50, 51]. In the present work, it was employed the gram-negative bacterium *L. pneumophila *to study the effect of propolis on NLRC4 inflammasome. Thus, BMDMs from C57BL/6 mice were treated with propolis for 1 hour and then infected with *L. pneumophila *for 12 hours. The results revealed that there was a reduction on IL-1*β* secretion by mouse macrophages that were infected but were pretreated with propolis (Figure 5(a)), suggesting that propolis can also modulate the NLRC4 inflammasome.

To investigate if the diminished amount of IL-1*β* found in BMDMs treated with propolis was not due to the antimicrobial activity of propolis on *L. pneumophila*, it was performed a CFU (colony forming units) assay in presence of propolis. We found that BMDMs treated with propolis and infected with *L. pneumophila *show no difference in the number of CFU found in macrophages in presence or absence of propolis (Figure 5(b)).

## 4. Discussion

Around 40% of medicines available nowadays were developed from natural sources: 25% from plants, 13% from microorganisms, and 3% of animals. From 1981 to 2002, 60% of medicines approved were natural products or obtained from these sources [52].

In this scenario, propolis is a very promising substance to be studied. It is a very complex compound from which many components have already been identified. Caffeic acid derivatives, flavonoids, and phenolic compounds, for instance, are usually found in many kinds of propolis [9, 32]. On the other hand, other substances are specific to a certain type of propolis, such as CAPE (Caffeic Acid Phenethyl Ester), commonly present in European propolis [53] and Artepillin C, a known exclusive biomarker of Brazilian green propolis [32, 54]. Although the active compounds of propolis are still under discussion, there are a large number of biological effects attributed to some specific components present in propolis, especially CAPE and artepillin C. Therefore, in the present work, these substances and some representatives of caffeic acid derivatives were investigated in the Brazilian green propolis standardized extract (EPP-AF).

Nevertheless, it is also known that natural products possess a synergistic effect resultant of the mix of their compounds and if the active compounds are not yet identified, the total extract is regarded as the “active principle” and, in that case, the biomarker compounds are used for quality control. Because of that, it is crucial that the first step to testing any propolis formulation is to standardize a propolis extract according to a reproducible chemical profile.

The results demonstrated that the chemical standardization of propolis prepared here corroborated with the propolis profile presented in our previous work, which already revealed an effective anti-inflammatory activity [9].

Ansorge et al. showed that cytokines produced by monocytes/macrophages (IL-1*β*, IL-12), as well as cytokines produced by Th2 type lymphocytes as IL-4, were found to be suppressed in the presence of propolis, whereas the production of TGF-*β*1 produced by regulatory T cells was ascertained to be increased [55]. These effects were mediated, at least in part, by caffeic acid phenethyl ester (CAPE) [55, 56], hesperidin, and quercetin [55]. The compound CAPE has been described to possess anti-inflammatory, antiproliferative, anticancerogenic, and antioxidant effects. Moreover, it has demonstrated to decrease IL-1*β* in bleomycin-induced pulmonary fibrosis in rats model [56, 57].

In the present work, it was verified that BMDMs pretreated with green propolis presented a reduction on IL-1*β* secretion after LPS and nigericin stimulation (Figure 3(a)). However, the results of chemical characterization of the green propolis standardized extract (EPP-AF) employed showed that CAPE is not present in the sample evaluated (Figure 2). In this regard, the next step that will be targeted is the evaluation of the standards found in the propolis extract used (Figure 1) in order to identify if one of them, specially the most interesting one, Artepillin C, is involved in the effect observed in the present work. Good results for Artepillin C are expected, fact suggested by the anti-inflammatory results presented for this compound by Paulino et al. [6]. We also verified that the reduction of IL-1*β* observed was not due to a cytotoxic effect of propolis on macrophages, since the cell viability assay with ethidium bromide showed that macrophages treated with 30 *µ*g/mL of propolis did not present pores in the plasmatic membrane even after 18 hours of treatment (Figures 3(b), 3(c), 3(d), 3(e), and 3(f)).

IL-1*β* is a very important proinflammatory cytokine. Once secreted, IL-1*β* mediates a variety of local and systemic responses to infection, such as induction of fever, promotion of T cell survival, B cell proliferation, and antibody production and mediates the transmigration of leukocytes [58]. Nevertheless, this cytokine is produced as inactive propeptides that need to be processed in order to be secreted from immune cells. The secretion of mature form of IL-1*β* occurs only after its cleavage by the protease caspase-1 and this process occurs in the presence of a molecular platform, named inflammasomes. Here we verified that modulation of IL-1*β* secretion by propolis was related with the modulation of the inflammasome, because there was a reduction on active caspase-1 stain in macrophages treated with propolis and stimulated with LPS and nigericin (Figure 4). It is important to mention that LPS plus nigericin is a classic activator of the NLRP3 inflammasomes. The NLRP3 inflammasome has been implicated in response to a broad spectrum of infectious agents, including the bacterial pathogens as *S. aureus, Vibrio cholerae, Escherichia coli, Neisseria gonorrhoeae, Chlamydia pneumoniae, and Citrobacter rodentium* [59–63]; the fungal pathogens *Candida albicans* and *Aspergillus fumigatus* [63–65]; viral pathogens such as influenza *A, encephalomyocarditis* virus, and vesicular stomatitis virus [66–68]; and the parasites *Schistosoma mansoni* and *Dermatophagoides pteronyssinus* [69]. Indeed, damage-associated molecular patterns (DAMPs) such as ATP, uric acid crystals, amyloid-*β* fibrils, hyaluronan, and microbial toxins all activate NLRP3 [42, 70, 71]. We also confirm that propolis inhibits the NLRP3 inflammasome by using ATP and gramicidin as NLRP3 activators, and found the same results (Figures 4(b), 4(c), and 4(d)).

Finally, we investigated the regulatory effect of propolis in other inflammasome already described: the NLRC4 inflammasome. Unlike the NLRP3 inflammasome, NLRC4 is currently thought to respond to gram-negative bacterial components. So, we used the gram-negative bacterium *L. pneumophila *to activate the NLRC4 inflammasome and also verified the reduction on IL-1*β* secretion by infected cells that were treated with propolis (Figure 5(a)). Regardless the putative role of propolis in NLRC4 inflammasome, our data clearly demonstrate that Brazilian green propolis extract acts in regulating the IL-1*β* secretion by NLRP3 inflammasomes. This is a remarkable finding, since it was demonstrated that mutations in inflammasome-related genes, such as NLRP3 and NLRP1, are associated with autoimmune and autoinflammatory disorders [72]. Therefore, the use of natural products such as Brazilian propolis may open promising therapeutic strategies for the treatment of these severe chronic autoinflammatory diseases.

## 5. Conclusion

Altogether, these data indicate that Brazilian green propolis extract (EPP-AF) reduces the secretion of IL-1*β* by inhibiting the inflammasome activation, thus contributing to explain the previously anti-inflammatory activities of propolis. This specific regulation can be important once IL-1*β* mediates a variety of local and systemic responses to infection and autoinflammatory disorders such as gouty arthritis and type II diabetes.

## Figures and Tables

**Table 1:** Chemical composition of propolis standardized extract (EPP-AF) after four years and half on shelf life stability conditions (mg/g) (*n* = 3).

Standards researched	Average ± SD	% CV
Caffeic acid	0.244 ± 0.001	0.520
*p*-Coumaric acid	1.475 ± 0.002	0.115
*trans*-cinnamic acid	0.138 ± 0.001	0.903
Aromadendrin	0.423 ± 0.001	0.177
Artepillin C	3.690 ± 0.016	0.431

SD: standard deviation.

% CV: coefficient of variation.
